# Sample Preparation Using Graphene-Oxide-Derived Nanomaterials for the Extraction of Metals

**DOI:** 10.3390/molecules25102411

**Published:** 2020-05-21

**Authors:** Natalia Manousi, Erwin Rosenberg, Eleni A. Deliyanni, George A. Zachariadis

**Affiliations:** 1 Laboratory of Analytical Chemistry, Department of Chemistry, Aristotle University of Thessaloniki, 54124 Thessaloniki, Greece; 2 Institute of Chemical Technology and Analytics, Vienna University of Technology, 1060 Vienna, Austria; egon.rosenberg@tuwien.ac.at; 3 Laboratory of Chemical and Environmental Technology, Department of Chemistry, Aristotle University of Thessaloniki, 54124 Thessaloniki, Greece; lenadj@chem.auth.gr

**Keywords:** graphene oxide, sample preparation, metal ions, food samples, environmental samples, biological samples, agricultural samples

## Abstract

Graphene oxide is a compound with a form similar to graphene, composed of carbon atoms in a sp^2^ single-atom layer of a hybrid connection. Due to its significant surface area and its good mechanical and thermal stability, graphene oxide has a plethora of applications in various scientific fields including heterogenous catalysis, gas storage, environmental remediation, etc. In analytical chemistry, graphene oxide has been successfully employed for the extraction and preconcentration of organic compounds, metal ions, and proteins. Since graphene oxide sheets are negatively charged in aqueous solutions, the material and its derivatives are ideal sorbents to bind with metal ions. To date, various graphene oxide nanocomposites have been successfully synthesized and evaluated for the extraction and preconcentration of metal ions from biological, environmental, agricultural, and food samples. In this review article, we aim to discuss the application of graphene oxide and functionalized graphene oxide nanocomposites for the extraction of metal ions prior to their determination via an instrumental analytical technique. Applications of ionic liquids and deep eutectic solvents for the modification of graphene oxide and its functionalized derivatives are also discussed.

## 1. Introduction

Among environmental pollutants, metals are of high importance due to their potential toxic effects and tendency to bioaccumulate in aquatic ecosystems. In small quantities, certain metals like iron, copper, cobalt, manganese, and zinc are nutritionally essential for a healthy life, while other metals such as mercury, arsenic, chromium, cadmium, and lead have no known role in biological systems and they are considered toxic and extremely dangerous even at trace levels [1,2,3]. The instrumental spectroscopic techniques commonly used for the determination of metals are flame atomic absorption spectroscopy (FAAS) [4], cold vapor atomic absorption spectroscopy (CVAAS) [5], electrothermal atomic absorption spectroscopy (ETAAS) [6], inductively coupled plasma optical emission spectrometry (ICP-OES) [7], and inductively coupled plasma mass spectrometry (ICP-MS) [8]. Metal ions are present in environmental samples at ultra-trace concentrations and, due to the existence of potential interferences and the complexity of matrices, the implementation of an extraction and preconcentration technique is required for their efficient determination [9,10].

A wide variety of novel sorbents including graphene, graphene oxide, carbon nanotubes, metal–organic frameworks, covalent organic frameworks, and zeolitic imidazole frameworks have been employed for the extraction of metal ions [11,12,13,14,15,16]. Those materials have been utilized in a wide range of analytical sample preparation techniques including dispersive solid phase extraction (d-SPE) [17], magnetic solid-phase extraction (MSPE) [18], pipette tip solid-phase extraction (PT-SPE) [19], solid phase microextraction (SPME) [20], stir bar sorptive extraction (SBSE) [21], etc.

Graphene is a non-polar hydrophobic carbon-based nanomaterial that was discovered by Geim et al. in 2004 [22]. Since then, graphene has attracted a lot of attention due to its extraordinary mechanical, thermal, structural, and electronic properties, as well as its high specific surface area. It consists of a single layer of carbon atoms densely packed in a honeycomb crystal lattice that forms graphite sheets. Applications of graphene include preparation of nanocomposites, heterogenous catalysis, drug delivery, gas storage, molecular probing, and electrochemical sensors [23,24,25,26,27,28,29,30]. However, graphene is insoluble and hard to disperse in most solvents because of strong intermolecular van der Waals interactions [31].

Graphene oxide (GO) is the oxidized form of graphene, which can be obtained from natural graphite powder through oxidation with an anhydrous mixture of sulfuric acid, sodium nitrate, and potassium permanganate [32]. Graphene and graphene oxide show a similar structure, which is composed of carbon atoms in sp^2^ hybridization linked within a single-atom layer [33,34,35]. GO is of more polar and hydrophilic character than graphene, since it contains a large number of oxygen-containing groups including hydroxyl, carboxyl, and epoxy groups. Graphene oxide sheets are negatively charged in aqueous solutions due to the ionization of carboxylic groups and since they contain oxygen atoms with a lone pair of electrons, they are ideal sorbents to bind metal ions both through ionic and coordinative interaction. The adsorbed metal ions can be subsequently eluted with the addition of acid with the H^+^ competing for the binding site [10,35,36].

Due to the two-dimensional plane structure of GO, the material has a high sorption capacity and is an excellent sorbent for solid-phase extraction. A limitation of graphene oxide is the significant π–π stacking interactions between the GO nanosheets, which lead to aggregation and restacking of the nanosheets. As a result, some active adsorption sites of the adsorbent are blocked and thus its specific surface area is reduced. Functionalization of graphene oxide can take place in order to prevent aggregation, improve its behavior in aqueous solutions, and enhance its selectivity towards the target metal ion and/or the extraction efficiency of the sorbent. Moreover, GO can form magnetic nanocomposites with Fe_3_O_4_ nanoparticles through electrostatic interactions between the negatively charged GO nanosheets and the positively charged surface of magnetite. The magnetic nanocomposites combine the high adsorption efficiency of GO and the convenience of magnetic separation of Fe_3_O_4_ nanoparticles [37,38,39,40,41,42,43,44].

Several reviews have been published regarding the applications of graphene and graphene-based sorbents in the field of sample preparation [45,46,47,48,49]. Herein, we aim to discuss the applications of graphene oxide and its functionalized derivatives for the extraction and preconcentration of metal ions from environmental, agricultural, biological, and food samples. Applications of ionic liquids (ILs) and deep eutectic solvents (DESs) for the modification of graphene oxide are also discussed.

## 2. Synthesis of Graphene-Oxide-Derived Materials

Hummers′ method with or without modification is the most common synthetic route for the preparation of graphene oxide. Typically, graphite is dispersed in sulfuric acid and the dispersion is stirred. Subsequently, potassium permanganate is added dropwise to prevent a temperature rise. The resulting brownish slurry is diluted in water and final addition of hydrogen peroxide takes place. The mixture is centrifuged and washed with hydrochloric acid and water. Finally, filtration and freeze-drying of the obtained GO material take place [32].

Magnetic graphene oxide (GO/Fe_3_O_4_) can be produced by various synthetic routes. The one-step chemical co-precipitation approach is the most common approach for the preparation of magnetic graphene oxide. In this case, graphene oxide is dispersed in water, salts of Fe^2+^ and Fe^3+^ are added in appropriate concentrations, and the mixture is heated under reflux. Subsequently, ammonia is added dropwise to precipitate the ferric and ferrous ions. Graphene oxide can be also prepared with the solvothermal approach or by subjecting a mixture of GO and Fe_3_O_4_ to stirring, mechanical shaking, or ultrasonic treatment [50,51,52,53].

**Figure 1:**
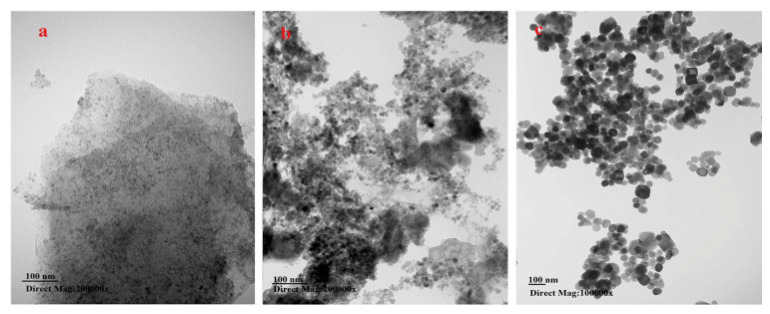
Transmission electron micrograph of magnetic reduced graphene oxide (RGO) prepared by the solvothermal (**a**), hydrothermal (**b**), and co-precipitation approach (**c**). Reproduced with permission from Reference [57]. Copyright Elsevier, 2015.

Reduced graphene oxide (RGO) is a nanomaterial obtained by chemical reduction of graphene oxide. RGO contains fewer oxygen groups than GO and reduction leads to an increase in the porosity as a result of exfoliation and rearrangement of layers [54]. RGO can be easily synthesized by dispersing GO in water and using hydrazine hydrate as a reductant. The reduction is normally carried out under stirring and heating. Magnetic RGO can be also prepared by approaches similar to those for the preparation of GO/Fe_3_O_4_, such as chemical co-precipitation, solvothermal, or hydrothermal approaches. In this way, the benefits of RGO and magnetic nanoparticles are combined to prepare highly efficient sorbents. Other reduction methods including electrochemical reduction, thermal reduction, microwave and photo reduction, photo-catalyst reduction, and reduction with green chemicals (e.g., ascorbic acid) can also be implemented for the preparation of RGO from GO [55,56,57]. Figure 1 shows the transmission electron microscopy (TEM) images of magnetic RGO prepared by the solvothermal (a), hydrothermal (b), and co-precipitation approaches (c).

Functionalization of graphene oxide can be employed in order to increase its potential applications. The functional modification of graphene oxide can not only maintain its excellent properties, but it also introduces new functional groups able to provide new characteristic to the sorbent. For this purpose, multifunctional organic materials such as polymers, nanoparticles, organic compounds, and multidentate chelating ligands have been examined. Various synthetic routes and various functional groups have been employed for the functionalization of GO and GO/Fe_3_O_4_ nanoparticles.

The selection of the functional group is based on the scope of the application (i.e., extraction of a metal ion, extraction of a complex compound of a metal ion, etc.), since different functional groups result in different characteristics. The modification of GO (e.g., with organic functional groups, such as amino, carboxyl, and mercapto groups) aims to enhance the selectivity of the sorbent towards the target analyte, the sensitivity of the determination, and the overall performance of the extraction procedure. Functionalization methods for graphene oxide mainly include covalent functionalization, non-covalent functionalization, and elemental doping.

Covalent functionalization involves combining graphene oxide with functional groups by forming covalent bonds in order to improve processability and introduce new functions to the sorbent. Covalent functionalization can be mainly divided into carbon-skeleton functionalization, hydroxyl functionalization, and carboxyl functionalization. On the other hand, non-covalent functionalization is based on π–π bond interaction, hydrogen bond interaction, ion interaction, and electrostatic interaction. In this approach, the structure and excellent properties of graphene oxide are maintained, while its dispersibility and stability are improved. Finally, element doping modifications are performed to incorporate different elements into the sorbent and thus enhance the overall performance of the material [58,59].

## 3. Extraction of Metal Ions with Graphene-Oxide-Derived Materials

The applications of graphene-oxide-derived materials for the extraction of metal ions are summarized in Table 1.

### 3.1. Extraction of Mercury

A polythiophene (PTh)-modified magnetic GO nanocomposite was synthesized and used for the determination of mercury in seafood prior to its detection by flow-injection cold vapor atomic absorption spectrometry (FI-CVAAS) [60]. Sulfur-containing molecules such as polythiophene exhibit good affinity towards mercury, which acts as a soft acid. As a result, functionalization aimed to enhance the selectivity of the extraction, as well as the stability of the magnetic GO nanoparticles. The developed method exhibited a low detection limit, satisfactory recovery and accuracy.

A magnetic graphene oxide modified with 2-pyridinecarboxaldehyde thiosemicarbazone groups (2-PTSC) was utilized for the preconcentration of trace amounts of mercuric ions from food (fish, rice, tea, milk) and environmental water samples followed by determination by ICP-OES [61]. Graphene oxide was functionalized with 2-pyridinecarboxaldehyde thiosemicarbazone ligands and the composite was then modified with magnetite nanoparticles via the co-precipitation approach. The 2-PTSC functional groups were chosen due to their favorable coordination capacity and selectivity for Hg(II).

Mercury has been also extracted from environmental water samples by a mercapto-grafted graphene oxide–magnetic chitosan (GO–MC) biosorbent [62]. Chitosan is a natural polymer that can improve the stability of magnetic GO nanocomposites, while exhibiting high adsorption capacity toward many types of metal ions. Functionalization with mercaptopropyltrimethoxysilane (MPTS) was chosen since MPTS is an effective ligand that contains a sulfur atom with good affinity towards soft metals like mercury. The nanocomposite was prepared through a chemical reaction of the amine group of magnetic chitosan with the carboxyl group of GO.

### 3.2. Extraction of Chromium

Kazemi et al. synthesized magnetic graphene oxide via the chemical co-precipitation approach and used it for the speciation of chromium ions by MSPE followed by FAAS determination [63]. The proposed method was implemented for the analysis of environmental water samples. Even though no functionalization was performed, good extraction efficiency was reported. Moreover, the sorbent was found to be reusable for at least 10 further applications.

Chromium species were extracted from tannery wastewater, electroplating wastewater, and river-water samples with a GO material decorated with triethylenetetramine-modified Fe_3_O_4_ nanoparticles followed by sequential speciation and determination by FAAS [64]. The surface functionalization was performed to prevent aggregation of the GO nanosheets and to enhance the extraction selectivity, since triethylenetetramine can form a complex with chromium. The nanocomposite was synthesized by the in situ coupling of triethylenetetramine with the carboxylic groups of graphene oxide using *N*,*N*’-dicyclohexylcarbodiimide as a coupling agent.

Sarikhani and Manoochehri synthesized an imidazolium-functionalized magnetite GO nanocomposite and used it for the determination of Cr(III) and Cr(VI) species by ETAAS [65]. For this reason, GO/Fe_3_O_4_ was synthesized and modified with a SiO_2_ layer, followed by further modification with imidazolium and thioamine moieties. The developed sorbent was successfully employed for the MSPE of chromium species from vegetable (leek, radish, radish leaves, fenugreek, parsley, beetroot, and basil) and wastewater samples. The presence of imidazolium rings enhanced the selectivity of the sorbent toward Cr(VI) ions via electrostatic interaction, while the presence of thioamine functional group enhanced its selectivity towards Cr(III) ions.

In 2016, Seidi and Majd synthesized a polyaniline (PANI)-functionalized magnetic graphene oxide nanocomposite and used it for the MSPE of Cr(VI) ions from environmental water samples prior to their determination by graphite furnace atomic absorption spectrometry (GFAAS). For this purpose, magnetic graphene oxide was prepared through the co-precipitation approach and was treated with cetyltrimethylammoniumbromide in water. Aniline and sodium persulfate as an oxidant were added to produce the desired sorbent. Due to anion-exchange interactions that were enhanced by the presence of PANI functional groups, the novel material showed high extraction efficiency towards the chromate ions [66].

### 3.3. Extraction of Cadmium

Cadmium was extracted from environmental water and rice samples using GO/Fe_3_O_4_ prior to its determination by FAAS [67]. The co-precipitation approach was employed for the synthesis of the sorbent. In order to enhance the extraction efficiency, an Alizarin red S (ARS) was employed as a chelating reagent. Due to the presence of quinoid oxygen with two hydroxyl groups, ARS can form stable complexes with metal ions. Moreover, ARS possesses a benzene ring structure and can therefore exhibits strong π-stacking interactions and hydrophobic interactions with GO, resulting in effective adsorption of the ARS complex with cadmium ions.

### 3.4. Extraction of Gold

Gold ions have been successfully extracted from environmental water samples using magnetic GO/Fe_3_O_4_ prior to their determination with flow injection flame atomic absorption spectrometry (FI-FAAS) [68] and microwave plasma-atomic emission spectrometry (MP-AES) [68]. In both cases, the magnetic nanocomposite was prepared through the chemical co-precipitation method. Good sorbent reusability was reported [69].

### 3.5. Extraction of Cobalt

Magnetic graphene oxide has been employed for the ligand-free extraction of cobalt from a wide variety of real samples including water, black tea, rice, wheat, orange, apple, saliva, and urine prior to its determination with ETAAS [70]. With the GO/Fe_3_O_4_ sorbent, an acceptable tolerance limit for potentially interfering ions was obtained.

### 3.6. Extraction of Zinc

In 2017, Babei et al. developed an MSPE method based on GO/Fe_3_O_4_@polythionine for the extraction of zinc from water and food samples prior to its determination by FAAS [71]. For the fabrication of the material, GO/Fe_3_O_4_ was mixed with polythionine and ferric chloride hexahydrate was used as a catalyst. Subsequently, hydrogen peroxide was dropwise added as an oxidizing material and the reaction mixture was heated at 50 °C for 1 h under stirring. The developed method showed good extraction efficiency, sensitivity, and repeatability and was successfully applied for the analysis of water, flour, celery, and egg samples.

Kazemi et al. synthesized a Zn(II)-imprinted polymer grafted onto a graphene oxide/magnetic chitosan nanocomposite and used it for the selective extraction of zinc ions prior to its determination by FAAS [72]. The synthesized ion-imprinted polymer showed high selectivity towards zinc, high adsorption capacity, and rapid mass transfer due to the extremely large surface area and multi imprinting sites of the magnetic chitosan/GO support. The developed sorbent was successfully employed for the selective extraction and determination of zinc in various samples including well water, drinking water, and food samples (black tea, rice, and milk).

### 3.7. Extraction of Copper

An amino-functionalized GO/Fe_3_O_4_ nanocomposite was also employed for MSPE of copper ions from food samples prior to their determination by FAAS [73]. For this purpose, GO was synthesized via Hummers’ method and was dispersed in ethylene glycol. Accordingly, ferric chloride and 1,6-hexadiamine were used to prepare the functionalized GO/Fe_3_O_4_ sorbent through a solvothermal approach. The functionalization of the sorbent was performed in order to enhance the extraction efficiency. The MSPE procedure was successfully employed for the analysis of eggplant, red lentil, and mushroom samples.

### 3.8. Extraction of Lead

Magnetic graphene oxide functionalized with 4-(2-pyridylazo)resorcinol (PAR) (GO–PAR@Fe_3_O_4_), was synthesized and applied for preconcentration of Pb(II) from environmental water and food samples prior to its determination by furnace atomic absorption spectrometry [74]. Therefore, GO–PAR was prepared from GO and PAR under reflux at 70 °C for 24 h, and then the GO–PAR@Fe_3_O_4_ sorbent was prepared with the co-precipitation method. Due to the presence of azo and OH functional groups of PAR, an increase in recovery in the extraction step was observed. The method was successfully applied for the preconcentration of lead from tap, well, and river water as well as rice and fruit juices.

Islam et al. developed a novel sorbent by coupling GO on chloromethylated polystyrene through an ethylenediamine spacer unit to develop an SPE method for the preconcentration/separation of lead prior to its determination by FAAS [75]. The polymer-bound GO restricts irreversible aggregation of the sorbent and its escape into the ecosystem, making the developed SPE method environmentally friendly. Finally, good extraction efficiency, reproducibility, and reusability were observed.

### 3.9. Extraction of Thallium

In 2017, Nazari et al. synthesized a GO/Fe_3_O_4_ conjugate to the surface of which a 4-methyl-2(2-pyrazinyl)-1,3-thiazole-5-carboxy acid (MPTCA) chelating agent was covalently linked and used it for sequential MSPE of thallium [76]. The chelator was employed to enhance the affinity of the sorbent towards Tl(III) ions in order to enhance the extraction efficiency. After complex formation, dispersive liquid phase microextraction of the thallium chelate took place, followed by determination by GFAAS. The combined sample preparation procedure exhibited high sensitivity, low LOD, and a high enhancement factor.

### 3.10. Extraction of Cerium

Farzin et al. synthesized magnetic reduced graphene oxide decorated with thioglycolic-acid-capped cadmium–tellurium (CdTe) quantum dots (QDs) and used it for the MSPE of Ce(III) from water samples prior to its determination by ICP-OES [77]. Manipulation of the surface chemistry of CdTe QDs by their capping with thioglycolic acid improved their selective behavior towards specific metal ions in complex sample matrices, since they can act like binding ligands. The designed sorbent provided low LOD and acceptable reusability, but it was costly. In order to prevent sorbent decomposition at the desorption step with the addition of acid, elution was performed with acetohydroxamic acid in sodium perchlorate and an evaporation step was required.

### 3.11. Extraction of Samarium

Samarium has been extracted from aqueous samples with 10-phenanthroline-2,9-dicarboxilic acid (PDA)-modified GO/Fe_3_O_4_ prior to its determination by ICP-OES [78]. The presence of PDA functional groups enhanced the selectivity of the extraction procedure, since carboxylic acids act as excellent ligand for the solvent extraction of lanthanides. As a result, the functionalized sorbent provided high extraction efficiency for samarium while it overcame the interference effects.

## 4. Multielement Extraction with Graphene-Oxide-Derived Materials

The applications of graphene-oxide-derived materials for multielement extraction are summarized in Table 2.

Non-functionalized graphene oxide was employed for the dispersive solid-phase extraction of Co(II), Ni(II), Cu(II), Zn(II), and Pb(II) prior to their determination by energy-dispersive X-ray fluorescence spectrometry (EDXRF) [79]. Good recovery, reproducibility, and extraction recovery were obtained. Graphene oxide has been also employed for the d-SPE of Cr(III), Co(II), Ni(II), Cu(II), Zn(II), and Pb(II) as their complexes with 2-(5-bromo-2-pyridylazo)-5-diethylaminophenol (5-Br-PADAP) using graphene oxide nanoparticles [80]. The chelation reagent does not form complexes with the alkali and alkaline earth metals, and it was therefore employed to enhance the selectivity of the extraction. In order to enhance the convenience of the d-SPE method, Ghazaghi et al. developed a coagulating homogenous extraction procedure based on coagulation of homogeneous GO solution with the aid of polyethyleneimine (PEI) [81]. In this work, PEI assisted the separation of the dispersed GO from the sample solution and provided satisfactory extraction recovery.

Solid-phase extraction of Co(II) and Ni(II) was performed by Pourjavid et al. using graphene oxide as adsorbent. In order to enhance the method’s selectivity, N-(5-methyl-2- hydroxyacetophenone)-N′-(2-hydroxyacetophenone) ethylene diamine (MHE) was used as a chelating agent and the complex of MHE and metal ions was extracted by graphene oxide in a SPE column [82].

Sitko et al. synthesized GO functionalized with spherical silica (GO@SiO_2_), coupling the amino groups of spherical aminosilica and the carboxyl groups of GO. The GO@SiO_2_ sorbent was packed into a SPE column and used for the extraction of Cu(II) and Pb(II) from water prior to their determination by FAAS [83]. Since small particles of GO can cause serious problems in SPE, such as high pressure in SPE system and the loss of adsorbent material, silica was covalently bonded with GO nanosheets to overcome these problems. Graphene-oxide–silica-composite-coated hollow fibers were synthesized and used for the online SPME of Mn(II), Co(II), Ni(II), Cu(II), Cd(II), and Pb(II) in environmental water samples prior to their determination by ICP-OES [84]. The novel fibers exhibited high adsorption capacity, reproducibility, and stability as well as long lifespan (more than 50 SPME cycles). Compared to the silica-coated hollow fiber, the GO–silica composite showed a different adsorption behavior, resulting in higher extraction efficiencies.

In order to combine the properties of GO with the ease in separation of magnetic nanoparticles, Sun et al. synthesized a magnetic graphene oxide nanocomposite with the one-step co-precipitation approach and used it for the MSPE of heavy metals from biological samples [85]. The GO/Fe_3_O_4_ sorbent was successfully employed for the extraction of Co(II), Ni(II), Cu(II), Cd(II), and Pb(II) from plasma and urine samples prior to their determination with ICP-MS.

Various polymers have been employed for the functionalization of GO, including polyaniline–polypyrrole (PANI-PPy) [86], polypyrrole–polythiophene (PPy-PTh) [87], and poly(vinylacetate-co-divinylbenzene) (DVB-VA) [88]. A polyaniline–polypyrrole-functionalized SiO_2_-coated magnetic graphene oxide composite was prepared and used for the MSPE of chromium and lead ions at trace levels in food and environmental water samples. Both polyaniline and polypyrrole contain amine and imine groups that can serve as good sorption sites for metals from complex matrices. Adsorption of metal ions to those polymers is based on hydrogen bonding π–π interactions, as well as ion-exchange interactions and chemical sorption. As a result, the adsorption capacity and the selectivity towards the target analytes was enhanced after functionalization [86].

**Figure 2:**
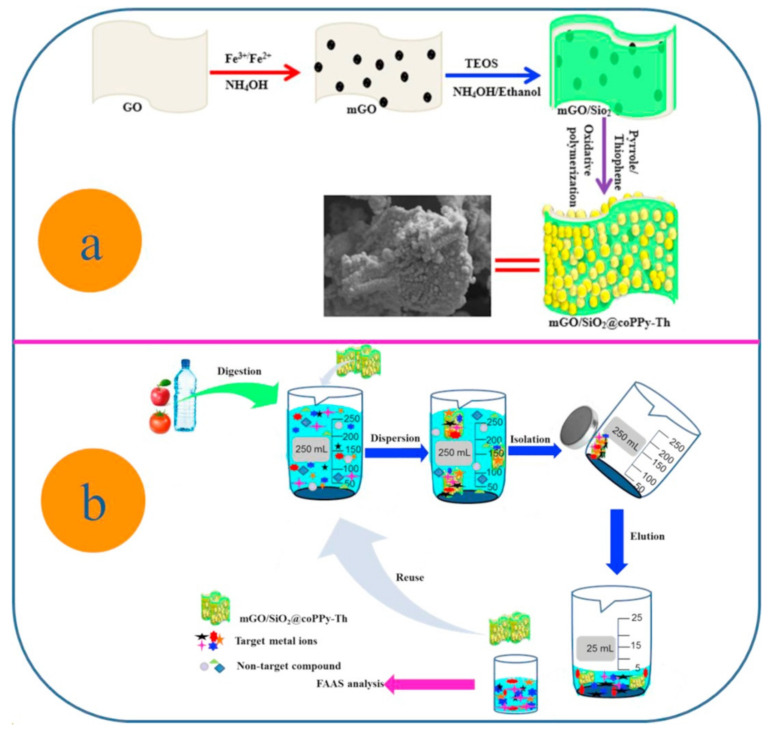
Synthesis (**a**) and application (**b**) of the polypyrrole–polythiophene functionalized magnetic GO. Reproduced with permission from Reference [87]. Copyright Elsevier, 2017.

In 2017, Molaei et al. synthesized a SiO_2-_coated magnetic GO modified with polypyrrole–polythiophene. For this purpose, magnetic GO was coated with SiO_2_ and the composite was modified by a copolymer with an in situ simultaneous oxidative polymerization of pyrrole and thiophene monomers, using iron chloride as an oxidant and dopant. The presence of N- and S-containing moieties enhanced the extraction efficiency of the nanocomposite and the selectivity of the MSPE procedure. The novel sorbent was successfully employed for the extraction of trace amounts of heavy metals (copper, lead, chromium, zinc, and cadmium) from water and agricultural samples prior to FAAS determination [87]. Figure 2 shows the synthesis (a) and application (b) of the PPy-PTh-functionalized magnetic GO.

A magnetic allylamine-modified GO–poly(DVB-VA) was synthesized and used for the MSPE of Pb(II), Cd(II), Cu(II), Ni(II), and Co(II) prior to their determination by FAAS. The method was successfully employed for the analysis of natural water and food samples. Due to the increase of the hydroxyl and carboxyl group functional groups, enhancement of extraction recoveries compared to the conventional GO/Fe_3_O_4_ sorbent was reported [88].

Ethylene diamine (EDA) [89] and diethylenetriamine (DETA) [89] are two examples of amines that were employed for the functionalization of GO. Ethylene diamine (EDA)-modified GO has been used for the d-SPE of Fe(III), Co(II), Ni(II), Cu(II), Zn(II), and Pb(II) from water samples. Due to the presence of the nitrogen-containing groups on the surface of the nanocomposite, no need for chelating reagents exists. These groups are able to bind with the metal ions to form a complex through sharing an electron pair [89]. Diethylenetriamine (DETA) has been also used for the functionalization of magnetic graphene oxide through the formation of amide linkage bonds between the amine groups of DETA and the oxygen-containing functional groups of GO. The functionalized nanocomposite was employed for the MSPE of lead and cadmium from water and vegetable samples prior to their determination by FAAS. Due to the strong chelating ability of the amine groups of DETA towards the target analytes, excellent adsorption capacity was observed [90].

Pytlakowska et al. synthesized a mercapto-functionalized GO via a direct reaction between in-situ-generated mixed phosphoric–carboxylic anhydrides and sodium sulfide with a GO surface. The sorbent was employed for the d-SPE of Cr(III), Cu(II), Zn(II), and Pb(II) from water samples prior to their determination by EDXRF, showing good selectivity towards the target analytes [91]. Magnetic graphene oxide modified with 2-mercaptobenzothiazole (MBT) was synthesized and used as a selective nanosorbent for MSPE of Au(III), Pd(II), and Ag(I) from water, ore, and automobile catalyst samples. For this purpose, graphene oxide was treated with MnFe_2_O_4_ nanoparticles to give the magnetic mag-GO sorbent, followed by modification with sodium dodecyl sulfate to create admicelles on the surface of the mag-GO. Finally, 2-mercaptobenzothiazole was immobilized on the sorbent surface as the metal chelating agent in order to improve the selectivity towards the platinum group metals (PGMs) [92]. Magnetic GO modified with 2-mercaptobenzothiazole (MBT) has been also used for the MSPE of Cd(II), Cu(II), and Pb(II) from water and vegetable samples prior to their determination by FAAS [93].

Sitko et al. synthesized a (3-mercaptopropyl)-trimethoxysilane (MPTMS)-functionalized graphene oxide nanocomposite and used it for the extraction of Co(II), Ni(II), Cu(II), As(III), Cd(II), and Pb(II) from water samples prior to their determination by total-reflection X-ray fluorescence spectrometry (TXRF). Due to the presence of the mercapto-functional groups, enhanced extraction efficiency was observed. Moreover, the nanocomposite exhibited good selectivity towards arsenite in the presence of arsenate and it can be therefore used for arsenic speciation [94].

Trithiocyanuric (TTC) acid [95] and 3-(1-methyl-1H-pyrrol-2-yl)-1H-pyrazole-5-carboxylic acid (MPPC) [96] are two examples of organic acids that have been employed for the functionalization of GO. Graphene oxide nanosheets were modified with trithiocyanuric acid for extraction and enrichment of Pb (II) and Cu (II) ions in seawater prior to its determination by FAAS. Trithiocyanuric acid was chosen as it is a robust chelating ligand for extracting divalent and univalent metal ions. Increased selectivity and adsorption capacity towards the target analytes were observed [95]. Pourjavid et al. synthesized a MPPC-functionalized GO nanocomposite and used it for the SPE of Mn(II) and Fe(III) from water, food, and biological samples prior to their determination by FAAS. Due to the presence of the chelating agent, the selectivity and adsorption efficiency were improved [96].

Other compounds that have been used for the functionalization of graphene oxide for the extraction of metal ions are multi-walled carbon nanotubes (MCNTs) [97], 8-Hydroxyquinoline (8-HQ) [98], glycine [99], and 1,5-bis(di-2-pyridil)methylene thiocarbonhydrazide (DPTH) [100].

Zhu et al. synthesized multi-walled carbon nanotubes (MCNTs) dispersed in GO colloids that were further functionalized with diethylenetriamine, and the resulting nanocomposite was used for the MSPE of Cr(III), Fe(III), Pb(II), and Mn(II) ions from waste water samples prior to their determination by ICP-OES. MCNTs exhibit high surface area, mechanical strength, and chemical stability. Compared to other SPE adsorbents, the reported nanocomposite combined the advantages of MCNTs and GO and exhibited high extraction efficiency towards the target analytes [97].

8-Hydroxyquinoline has been employed for the functionalization of magnetic graphene oxide in order to enhance its selectivity. 8-HQ is a nitrogen-heterocyclic aromatic hydrocarbon and a strong bidentate chelating agent. The functionalized sorbent was used for the MSPE of Cd(II) and Pb(II) ions from tomato, mushroom, lettuce, lipstick, and fish samples prior to their determination by FAAS. It was concluded that after functionalization with 8-HQ, the GO/Fe_3_O_4_ nanoparticles could be used for the extraction of cadmium and lead ions from complex matrices with high selectivity. Compared with the non-functionalized GO/Fe_3_O_4_ sorbent, the magnetic 8-HQ modified GO nanocomposite exhibited higher adsorption capacities towards the target analytes [98].

Glycine has been also employed for the functionalization of GO/Fe_3_O_4_. The wrinkled structure of GO–Gly shows a larger surface for a single element and high extractive capacity. Moreover, the presence of donating atoms in the glycine structure could influence the selectivity of the sorbent towards different metal species by influencing the affinity of metal species for sorption sites of the functionalized GO/Fe_3_O_4_. The sorbent was used for the extraction of Cr(III), Zn(II), and Cu(II) ions from water samples prior to energy-dispersive X-ray fluorescence spectrometric determination [99].

The noble metals Sb and Hg have been extracted from water samples using functionalized magnetic graphene oxide prior to their determination by on-line ICP-OES. For this purpose, graphene oxide was functionalized with 1,5-bis(di-2-pyridyl)methylene thiocarbohydrazide (DPTH) chemically linked to iron oxide nanoparticles in order to enhance sorbent selectivity [100].

Heavy metals and rare earth elements have been extracted from environmental samples with graphene oxide–TiO_2_. The modification of GO with titania enhanced the sorbent stability and the extraction efficiency towards the target ions. The novel sorbent was immobilized in a microcolumn. The proposed method was implemented for the extraction of metal ions and REEs prior to their determination by ICP-OES, and it showed good anti-interference capability. Moreover, the novel SPE column was found to be reusable for more than 90 applications [101]. In 2014, Su et al. fabricated a Fe_3_O_4_@SiO_2_@polyaniline–GO nanocomposite and used it for the extraction of rare earth elements in tea leaves and environmental water samples prior to their determination with ICP-MS. Polyaniline enhanced the stability and the adsorption sites of magnetic GO, providing high extraction efficiency. The developed method was able to provide a high enhancement factor, low detection limits, and wide linear range, as well as sufficient tolerance towards potentially interfering ions [102]. Chemically modified graphene oxide was also used for the extraction of REEs from nuts and water samples prior to their determination by ICP-MS. GO was oxidized and the increased number of oxygen-containing active sites resulted in higher extraction efficiency compared to GO and reduced GO sorbents. The d-SPE method exhibited good accuracy and reproducibility and the oxidized graphene oxide (OGO) sorbent was found to be reusable for at least 12 applications [8].

## 5. Application of Ionic Liquids and Deep Eutectic Solvents for the Modification of GO

Ionic liquids are an alternative to environmentally harmful ordinary organic solvents that are gaining more and more popularity lately [103]. These materials are generally composed of bulky, nonsymmetrical organic cations including imidazolium, pyrrolidinium, pyridinium, ammonium, or phosphonium and different inorganic or organic anions such as tetrafluoroborate or bromide anions [104]. In the field of analytical chemistry, ionic liquids have been used as ionic-liquid-supported membranes, as additives in mobile phases, as surface-bonded stationary phases, and as extraction solvents for sample preparation [104,105,106,107]. ILs have a tunable nature and their properties can be optimized through the choice of their cationic and anionic constituents. Among their extraordinary chemical and physical properties are negligible vapor pressure, excellent thermal stability, tunable viscosity, good miscibility with organic solvents and water, and their good extraction efficiency of metal ions and organic compounds [103,108]. The combination of ionic liquids and graphene-oxide-derived materials makes it possible to design and develop new extraction adsorbent phases with outstanding properties [103]. The applications of ILs as modifiers of GO-based materials for the extraction of metal ions are summarized in Table 3.

Alvand and Shemirani synthesized core–shell-structured GO/Fe_3_O_4_ nanospheres via covalent bonding and used it for ferrofluid d-SPE of Cd from environmental water samples, carrot, lettuce, and tobacco [109]. For this purpose, acetic acid was used as stabilizing agent and the acetic-acid-coated magnetic nanospheres were dispersed in 1-ethyl-3-methylimidazolium tetrafluoroborate. For the extraction procedure, the sample and 1-(2-pyridylazo)-2-naphtol (PAN) (complexing agent) were placed in a tube into which the ferrofluid was rapidly injected with the assistance of a syringe. The Cd-PAN complex was extracted, the sorbent was isolated with a magnet, and the aqueous phase was discarded. Elution was performed with nitric acid and the target analyte was finally determined by FAAS. The sorbent exhibited high recovery and high preconcentration factor.

In 2016, Lotfi et al. synthesized a covalently bonded double-charged ionic liquid on magnetic graphene oxide as and used it for the ultrasound-assisted MSPE of heavy metals from medicine capsules [110]. For this purpose, GO/Fe_3_O_4_ was modified with (3-mercaptopropyl)trimethoxysilane and 1,4-diazoniabicyclo[2.2.2]octane chloride. Due to the modification with a double-charged IL, the novel sorbent exhibited high adsorption capacity and was successfully used for the extraction of Pb(II), Cd(II), Ni(II), Cu(II), and Cr(III) prior to their determination by FAAS.

Aliyari et al. modified GO/Fe_3_O_4_ using 1-hexadecyl-3-methylimidazolium chloride via an electrostatic self-assembly technique [111]. For this purpose, immobilization of the dimethylglyoxime ligand onto magnetic nanoparticles coated with the surface-active IL, 1-hexadecyl-3-methylimidazolium chloride was carried out. The novel sorbent was successfully applied for the extraction of nickel from sea and river water, tea, spinach, cacao powder, and cigarette samples.

An amino-acid-derived ionic liquid was used for the coating of Fe_3_O_4_–SiO_2_–GO nanoparticles. The sorbent was employed for the extraction of Al(III), Cr(III), Cu(II), and Pb(II) from environmental water samples prior to their determination by ICP-OES [112]. The resulting ionic-liquid-functionalized nanocomposite was environmentally friendly, biocompatible, and showed obvious chelation with metal ions, resulting in low detection limits and high extraction recovery.

Rofouei et al. synthesized a magnetic bucky gel by mixing GO/Fe_3_O_4_ and 1-butyl-3-methylimidazolium hexafluorophosphate and used it for the preconcentration of Cu(II), Zn(II), Cd(II), Cr(III), Pb(II), and Co(II) ions from environmental water samples [113]. The term “bucky gel” was initially used to describe gelatinous composite materials composed of carbon nanotubes and ionic liquids. A bucky gel consisting of Fe_3_O_4_ nanoparticles, graphene oxide, and ionic liquid is an efficient sorbent for extraction of heavy metal ions from water prior to their determination by ICP-OES. The metal ions were complexed with 1-(2-pyridylazo)-2-naphthol and were further extracted by the sorbent. Subsequently, elution with acetonitrile was performed and the analytes were determined by ICP-OES. Compared with the free IL and GO/Fe_3_O_4_, the bucky gel showed higher extraction efficiency, indicating the appearance of a synergic effect in the IL/magnetic GO material.

**Figure 3:**
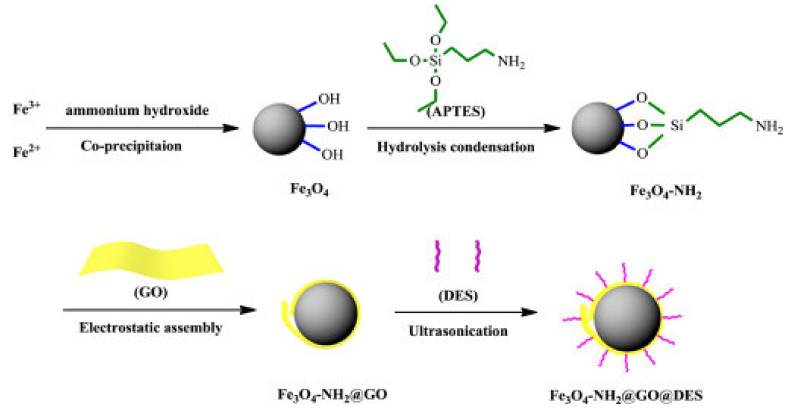
Preparation of GO nanoparticles modified with DES. Reproduced with permission from Reference [115]. Elsevier. Copyright Elsevier, 2016.

Deep eutectic solvents (DESs) are systems formed from a eutectic mixture of Lewis or Brønsted acids and bases that contain a variety of anionic and/or cationic species [114]. A DES typically consists of substituted quaternary ammonium salts and hydrogen bond donors. The most widely used salt is choline chloride (ChCl), since it is low-cost and can be derived from biomass [115]. They contain large, nonsymmetrical ions that have low lattice energy, resulting in low melting points. Although DESs and ILs show similar physical properties, their chemical properties suggest significantly different applications [114]. An example of a synthetic route that results in a magnetic GO modified with a DES is shown in Figure 3.

Due to their superior characteristics, such as low cost, easy preparation, low toxicity, tunability, low vapor pressure, biocompatibility, and biodegradability, DESs have a plethora of applications in various research areas. Among their applications are gas adsorption, biotransformation, purifying and manufacturing biodiesel, and metal-processing applications [114,116].

DESs exhibit an ionizable nature and strong hydrogen bonding, providing multiple benefits compared to traditional organic solvents. In the field of analytical chemistry, deep eutectic solvents have been used as a stationary phase for chromatography as well as mobile phases. DESs have been also successfully employed for microextraction of target analytes, modification of nanoparticles, or as eluents in sample preparation of various samples [117].

Magnetic graphene oxide nanoparticles have been treated with DESs in order to create new efficient adsorbents. A ternary hydrosulphonyl-based deep eutectic solvent comprised of choline chloride/itaconic acid/3-mercaptopropionic acid (molar ratio 2:1:1) was used to functionalize GO/Fe_3_O_4_ nanoparticles. The sorbent was employed for the removal of mercury ions from wastewater samples. Due to the presence of –SH groups, extraction efficiency was significantly improved compared with the non-functionalized magnetic graphene oxide sorbent. Moreover, the functionalized sorbent exhibited good stability, since it was found to be reusable for at least 7 further applications after regeneration [118].

## 6. Conclusions

Due to their significant surface area and high mechanical and chemical stability, graphene-oxide-derived nanocomposites have been successfully employed for the extraction of a wide variety of metal ions from environmental, food, biological, and agricultural samples. Graphene oxide contains a significant number of oxygen-containing functional groups that are able to bind with multivalent metal ions through both electrostatic and coordinate approaches. Magnetic composites of GO with Fe_3_O_4_ nanoparticles have been also evaluated in order to take advantage of the ease in magnetic separation of Fe_3_O_4_ and the high extraction efficiency of GO.

The main drawback of these sorbents is the potential aggregation and restacking of the nanosheets, which might lead to blocking of the active adsorption sites of the sorbent. In order to overcome this limitation, a wide variety of functional groups have been proposed and evaluated. Functionalization has also been found to enhance the dispersibility of the sorbent, as well as the extraction efficiency of the sorbent and/or selectivity towards the target analytes.

Finally, in order to enhance the extraction efficiency of the sorbent, modification of graphene oxide with ionic liquids and deep eutectic solvents has been proposed. In this case, the modification process is rapid, simple, and it does not require toxic organic solvents. The stability of the modified nanocomposites and their potential reusability are still relatively limited. However, compared with other novel sorbents such as metal–organic frameworks and zeolitic imidazole frameworks, GO nanocomposites exhibit higher reusability, meeting the requirements of green chemistry.

Since most applications of graphene-oxide-derived materials are focused on MSPE and d-SPE, future advances include the extension of the application of these materials to more and alternative sample preparation formats, including PT-SPE and SBSE. Moreover, evaluation of more functional groups in order to enhance the extraction efficiency and selectivity of the extraction process should be performed. It can be concluded that with the new and the anticipated developments in the field of graphene-oxide-derived nanomaterials, it is expected that these will become a useful tool in the analytical repertoire for trace inorganic analysis in environmental, biological, and food applications.

## Figures and Tables

**Table 1:** Application of graphene-oxide-derived materials for the extraction of metal ions.

Analyte	Sample Matrix	Sorbent	Functional Groups	Analytical Technique ^1^	LODs (µg L^−1^)	Adsorption Time (min)/ Desorption Time (min)	Recovery (%)	Adsorption Capacity (mg g^−1^)	Reusability	Ref.
Hg(II)	Seafood	GO/Fe_3_O_4_	Polythiophene	FI-CVAAS	0.03	21/2	85	1		[60]
	Fish, rice, tea, milk	GO/Fe_3_O_4_	2-Pyridinecarboxaldehyde	ICP-OES	0.008	3/4	97	NA		[61]
	Water	GO/Fe_3_O_4_	Chitosan, Mercaptopropyltrimethoxysilane	CVAAS	0.06	10/10	>95	>400		[62]
Cr(VI) & Cr(III) species	Water	GO/Fe_3_O_4_		FAAS	0.1	>5 min/3	97–103	60	At least 10 times	[63]
	Water	GO/Fe_3_O_4_	Triethylenetetramine	FAAS	1.4–1.6	30/-	>96	9.6–16.4		[64]
	Water	GO/Fe_3_O_4_	Imidazolium, thioamine	GFAAS	1.2 × 10^−3^	9/16.5	>95	304 (total)		[65]
Cr(VI)	Water	GO/Fe_3_O_4_	Polyaniline	GFAAS	5 × 10^−3^	20/4	68	14.8		[66]
Cd(II)	Water, rice	GO/Fe_3_O_4_		FAAS	0.21 × 10^−3^	2/1	>95	11.1		[67]
Au(II)	Water	GO/Fe_3_O_4_		FI-FAAS	4 × 10^−3^	Rapid/40 s	98–102	9.8	At least 10 times	[68]
	Water	GO/Fe_3_O_4_		MP-AES	5 × 10^−3^	10/5	97–101	192.1	Up to 20 times	[69]
Co(II)	Water, food, biological samples	GO/Fe_3_O_4_		ETAAS	0.02	½	70–106	60		[70]
Zn(II)	Water, food	GO/Fe_3_O_4_	Polythionine	FAAS	0.08	7/-	>87		At least 5 times	[71]
	Water, food	GO/Fe_3_O_4_	Chitosan, Zn-imprinted polymer	FAAS	0.09	10/5	>96	71.4	At least 9 times	[72]
Cu(II)	Eggplant, red lentil and mushroom	GO/Fe_3_O_4_	1,6-Hexadiamine	FAAS	0.9	10/2	>97		Up to 5 times	[73]
Pb(II)	Water, food	GO/Fe_3_O_4_	4-(2-pyridylazo)resorcinol	ETAAS	0.18 × 10^−3^	-/3	>98	133		[74]
	Water, food	GO	Polystyrene	FAAS	2.5	Not applicable	>99	227.9	Up to 50 times	[75]
Tl(III)	Water	GO/Fe_3_O_4_	4-methyl-2(2-pyrazinyl)-1,3-thiazole-5-carboxy acid	GFAAS	12 × 10^−3^	8/3	65	20.0		[76]
Ce(III)	Water	RGO/Fe_3_O_4_	Thioglycolic-acid-capped Cadmium–tellurium quantum dots	ICP-OES	0.1	10/6	>96	56.8	At least 12 times	[77]
Sa(III)	Water	GO/Fe_3_O_4_	10-phenanthroline-2,9-dicarboxilic acid	ICP-OES	1.4	20/12	>97			[78]

^1^ FI-CVAAS: Flow-injection cold vapor atomic absorption spectrometry, ICP-OES: Inductively coupled plasma optical emission spectrometry, CVAAS: Cold vapor atomic absorption spectrometry, FAAS: Flame atomic absorption spectroscopy, GFAAS: graphite furnace atomic absorption spectrometry, FI-FAAS: Flow-injection flame atomic absorption spectroscopy, MP-AES: Microwave plasma-atomic emission spectrometry, ETAAS: Electrothermal atomic absorption spectroscopy.

**Table 2:** Applications of graphene-oxide-derived materials for multielement extraction.

Analytes	Sample Matrix	Sorbent	Modification	Analytical Technique ^1^	LODs (µg L^−1^)	Adsorption Time (min)/ Desorption Time (min)	Recovery (%)	Adsorption Capacity (mg g^−1^)	Reusability	Ref.
Co(II), Ni(II), Cu(II), Zn(II), Pb(II)	Water	GO		ICP-OES	0.5–1.8	5/-	94–106	294–1119		[79]
Cr(III), Co(II), Ni(II), Cu(II), Zn(II), Pb(II)	Water	GO		EDXRF	0.07–0.25	15/-	94–104			[80]
Cr(III), Cd(II), Pb(II)	Water, saliva, urine	GO		ETAAS	5-12 × 10^−3^	Few seconds/-	94–103			[81]
Co(II), Ni(II)	Water, black tea, tomato	GO		FAAS	0.18–0.25	Not applicable	>95	6.8–7		[82]
Cu(II), Pb(II)	Water	GO	SiO_2_	FAAS	0.08–0.27	Not applicable	>95	6.0–13.6	At least 50 times	[83]
Mn(II), Co(II), Ni(II), Cu(II), Cd(II), Pb(II)	Water	GO	Silica	ICP-MS	0.39–22 × 10^−3^	5/1	85–119	4.6–25	At least 50 times	[84]
Co(II), Ni(II), Cu(II), Cd(II), Pb(II)	Plasma, Urine	GO/Fe_3_O_4_		ICP-MS	0.02–0.40	7/7	81–113	1.3–9.7	At least 20 times	[85]
Cr(III), Pb(II)	Rice, milk, wine, water	GO/Fe_3_O_4_	Polyaniline–polypyrrole, SiO_2_	ICP-MS	3.4–4.8 × 10^−3^	6.3/3.7	96–103	188.9–213.3	At least 6 times	[86]
Cu(II), Pb(II), Zn(II), Cr(III), Cd(II)	Water, agricultural samples	GO/Fe_3_O_4_	Polypyrrole–polythiophene, SiO_2_	FAAS	0.15–0.65	6.5/12	90–106	80–230	At least 5 times	[87]
Pb(II), Cd(II), Cu(II), Ni(II), Co(II)	Water, food samples	GO/Fe_3_O_4_	Poly(vinylacetate-co-divinylbenzene)	FAAS	0.37–2.39	-/-	>95			[88]
Fe(III), Co(II), Ni(II), Cu(II), Zn(II),Pb(II)	Water	GO	Ethylene diamine	EDXRF	0.06–0.1	5/-	>90			[89]
Cd(II), Pb(II)	Water, vegetables	GO/Fe_3_O_4_	Diethylenetriamine (DETA)	FAAS	0.38–0.40	10/2	>99	59.9–172.4		[90]
Cr(III), Cu(II), Zn(II), Pb(II)	Water	GO/Fe_3_O_4_	Mercapto-groups	EDXRF	0.06–0.10	10/-	>95	191.5–487.3		[91]
Au(III), Pd(II), Ag(I)	Water, ore and automobile catalyst	Magnetic GO	2-mercaptobenzothiazole	ICP-OES	0.05–0.08	10/3	90–103	28–45	At least 5 times	[92]
Cd(II), Cu(II), Pb(II)	Water, vegetables	GO/Fe_3_O_4_	2-mercaptobenzothiazole	FAAS	0.19–0.35	4/5	>99	156–179		[93]
Co(II), Ni(II), Cu(II), As(III), Cd(II), Pb(II)	Water	GO	(3-mercaptopropyl)-trimethoxysilane	TXRF	0.05–9.11	10/2	>94	18.1–108.3		[94]
Pb(II), Cu(II)	Water	GO/Fe_3_O_4_	Trithiocyanuric acid	FAAS	0.13–0.32	Not applicable	>90	0.46–0.75		[95]
Mn(II), Fe(III)	Water, food and biological samples	GO	3-(1-methyl-1H-pyrrol-2-yl)-1H-pyrazole-5-carboxylic acid	FAAS	0.31–355	Not applicable	>95	21.6–24.0		[96]
Cr(III), Fe(III), Pb(II), Mn(II)	Wastewater	GO	Multi-walled carbon nanotubes -DETA	ICP-OES	0.16–0.50	Not applicable	>95	5.4–13.8		[97]
Cd(II), Pb(II)	Vegetables, fish, lipstick	GO/Fe_3_O_4_	8-Hydroxyquinoline	FAAS	0.09–0.27	5/5	>96	133–150		[98]
Cr(III), Zn(II), Cu(II)	Water	GO/Fe_3_O_4_	Glycine	EDXRF	0.07–0.15	10/-	>97			[99]
Noble metals, Sb(III), Hg(II)	Seawater	GO/Fe_3_O_4_	1,5-bis(di-2-pyridyl)methylene thiocarbohydrazide	ICP-OES	0.05–2.60	Not applicable	90–106	4.5–9.7		[100]
Cu (II), Pb(II), La(III), Ce(III), Eu(III), Dy(III), Yb(III)	Water	GO	TiO_2_	ICP-OES	0.13–2.64	Not applicable	>90	0.8–13.5	At least 90 times	[101]
REEs	Water	GO/Fe_3_O_4_	Polyaniline, SiO_2_	ICP-MS	0.04–1.49 × 10^−3^	2/5	80–121	7.7–16.3	At least 30 times	[102]
REEs	Nuts, water	Oxidized GO		ICP-MS	0.03–1.8	15/1	60–90	6.1–12.2	At least 12 times	[8]

^1^ ICP-OES: Inductively coupled plasma optical emission spectrometry, EDXRF: Energy-dispersive X-ray fluorescence spectrometry, ETAAS: Electrothermal atomic absorption spectroscopy, FAAS: Flame atomic absorption spectroscopy, ICP-MS: Inductively coupled plasma mass spectrometry, TXRF: Total-reflection X-ray fluorescence spectrometry.

**Table 3:** Applications of ILs for the modification of GO-based materials for the extraction of metal ions.

Analyte	Sample Matrix	Sorbent	Ionic Liquids	Analytical Technique ^1^	LODs (µgL^−1^)	Adsorption Time (min)/ Desorption Time (min)	Recovery (%)	Adsorption Capacity (mg g^−1^)	Ref.
Cd(II)	River and seawater, carrot, lettuce and tobacco	GO/Fe_3_O_4_	1-ethyl-3-methylimidazolium tetrafluoroborate	FAAS	0.12	Few seconds/1 min	98–102	33.7	[109]
Pb(II), Cd(II), Ni(II), Cu(II) and Cr(III)	Medicine capsules	GO/Fe_3_O_4_ modified with (3-mercaptopropyl)trimethoxysilane	1,4-diazabicyclo [2.2.2]octane	FAAS	0.2–1.8	4 min/1 min	95–102	18.1–47.6	[110]
Ni(II)	Sea and river water, tea, spinach, cacao powder, cigarette	GO/Fe_3_O_4_	1-hexadecyl-3-methylimidazolium chloride	FAAS	0.16	15 min/2 min	97–99	129.9	[111]
Al(III), Cr(III), Cu(II), Pb(II)	Environmental water	Fe_3_O_4_-SiO_2_−GO	N-(3- Dimethylaminopropyl)-N-ethylcarbodiimide hydrochloride	ICP-OES	0.5–30 × 10^−3^	5 min/3 min	89–118	5.0–11.7	[112]
Cu(II), Zn(II), Cd(II), Cr(III), Pb(II) and Co(II)	Environmental water	GO/Fe_3_O_4_	1-butyl-3-methylimidazolium hexafluorophosphate	ICP-OES	0.1–1	10 min/6 min	34–94	312.5	[113]

^1^ FAAS: Flame atomic absorption spectroscopy, ICP-OES: Inductively coupled plasma optical emission spectrometry.
